# Inter‐brain synchrony is associated with greater shared identity within naturalistic conversational pairs

**DOI:** 10.1111/bjop.12743

**Published:** 2024-10-26

**Authors:** Neal S. Hinvest, Chris Ashwin, Muhammad Hijazy, Felix Carter, Chiara Scarampi, George Stothart, Laura G. E. Smith

**Affiliations:** ^1^ Department of Psychology University of Bath Bath UK; ^2^ Faculty of Business and law University of Northampton Northampton UK; ^3^ Department of Psychology University of Bristol Bristol UK; ^4^ Centre for the Interdisciplinary Study of Gerontology and Vulnerability (CIGEV) University of Geneva Geneva Switzerland; ^5^ Swiss National Center of Competences in Research LIVES – Overcoming Vulnerability: Life Course Perspectives Geneva and Lausanne Switzerland

**Keywords:** connectedness, EEG, inter‐brain synchrony, natural social interaction, neural synchrony, social behaviour, social identity

## Abstract

Inter‐brain synchrony occurs between individuals who feel connected socially, but how synchrony relates to felt connectedness under naturalistic social interaction has remained enigmatic. We hypothesized that inter‐brain synchrony between naturally interacting individuals might be associated with the internalization of a social identity, a link between an individual's personal identity and the social group to which the individual belongs. A convenience sample of sixty participants were split into dyads and interacted naturalistically on a social task. Through mapping EEG oscillatory waveforms onto a conceptual model categorizing the formation of a social identity within a naturalistic conversation, greater inter‐brain synchrony was observed in the emergent stage within the formation of a social identity compared to earlier stages, where a social identity was not present. We provide evidence for greater neural synchrony related to higher socio‐psychological connectedness during the development of social identity under naturalistic social interaction.

## BACKGROUND

The idea that shared psychological states have a critical influence on social behaviour is pervasive within social psychology. For example, in her seminal theory of social contagion, Elaine Hatfield posited that people unconsciously mimic the observed emotional state of others, leading to ‘social convergence’, which is a primary stage in developing appropriate empathic responses and social cohesion (Hatfield et al., 1993). Now, integration of methodological advances in social neuroscience, predominantly hyper‐scanning (the concurrent measurement of neurophysiological signals within two interacting individuals), and mathematical classification models, are enabling new insights into collective behaviour.

Inter‐brain synchrony (IBS), a phenomenon where brain activity patterns between two or more individuals become aligned or coordinated, has been observed with multiple methods, including *f*MRI, *f*NIRS and EEG, in a wide range of social interactions including joint attention (Szymanski et al., 2017), motor coordination (Müller & Lindenberger, 2022) and interactive decision‐making (Hu et al., 2018). This challenges the single‐person account of consciousness, in which consciousness is considered as first‐person and private (Valencia & Froese, 2020). With inter‐brain synchrony being observed in field studies (Dikker et al., 2017), attention has turned to its practical applications, including team‐based performance (Reinero et al., 2020; Réveillé et al., 2024) and brain–computer interface (BCI) (Nazneen et al., 2022).

Contemporary studies have moved out of the lab to more naturalistic environments to explore the relationship between inter‐brain synchrony and complex social psychological behaviours. For example, researchers have run a naturalistic social EEG experiment involving 12 high school students wearing wireless EEG headsets during 11 classes within a three‐month period. Inter‐brain synchrony in oscillatory waveforms between students was positively associated with self‐reported levels of both classroom engagement and social closeness reported by the students towards their teacher (Dikker et al., 2017). Reinero et al. (2020) used a similar technique to investigate inter‐brain synchrony between individual undergraduate students within teams of four completing problem‐solving tasks, with findings revealing that inter‐brain synchrony between the team members predicted team performance (Reinero et al., 2020). Together, these findings demonstrated that inter‐brain synchrony indexed within EEG oscillatory waveforms can be associated with shared social experiences, and, additionally, the viability of using modern, portable, EEG equipment to record neuro‐physiological measurements within naturalistic situations.

The current research furthers the work in this scientific domain by proposing that the neural synchrony found because of a shared social experience may represent the internalization of a shared social identity. A social identity is the component of an individual's self‐concept created from the social groups (categories) to which they perceive themself as belonging, while social identification is a person's felt connectedness to that group or category (Tajfel & Turner, 1979). Social identification is a multi‐faceted construct that includes both cognitive and affective components (Leach et al., 2008). Social identities and associated social identifications are constructed through interaction with others when people's emotions, cognitions and opinions become collectively shared and self‐defining (Bliuc et al., 2007; Haslam et al., 2003; Smith, Thomas, & McGarty, 2015). When individuals internalize a social identity, identify with the group, and that identity is salient, they are likely to conform to the behavioural norms of that group. The internalization of a social identity can be indexed through the language that people use (Pennebaker et al., 2003, 2015). For example, increases in extremist vernacular and linguistic markers are associated within within‐person changes in identification with extremist groups (Smith et al., 2020).

In this way, shared social identification can explain and predict social behaviour. Therefore, identifying a neural signature of the development of shared social identity could help facilitate the prediction of people's decisions and behaviours, potentially before they are even consciously aware of this process or their decision to act in line with group norms. Identifying this signature would have a significant range of applications as mentioned, notably within brain–computer interface (BCI) and team‐based performance.

Identifying a neural signature of social identity development will also contribute novel methods that more effectively align with the nature of the psychological process than do traditional, survey‐based methods. Within social psychology, there is a disconnection between the phenomena related research attempts to understand (which are inherently social, real‐time, and involve both conscious and unconscious processes), and the traditional measures (which are inherently individualistic, post‐hoc, and predominantly conscious) that are used to capture the phenomena associated with shared social identity. Hyper‐scanning is a methodological approach which can help answer many outstanding questions within social psychology that are difficult to study using the more traditional methods (Babiloni & Astolfi, 2014; Kasai et al., 2015; Koike et al., 2015).

To map the emergence of a shared social identity, we used a naturalistic method where freely interacting participants that did not initially know each other were tasked with reaching a consensus on a topical matter. The current study used an inter‐disciplinary, exploratory approach where dyads wearing EEG headsets discussed a contemporary issue after being instructed to attempt to come to agreement on actions that people like them could take regarding this issue. Inter‐brain synchrony was measured via dynamic time warping (DTW), a mathematical algorithm that quantifies the similarity between two temporal signals. The degree to which a social identity was being formed within the dyad was indexed using the identity‐norm nexus (INN‐)formation model of Smith, Gavin, and Sharp (2015) and Smith, Thomas, and McGarty (2015), which describes four stages in which individuals establish a shared social identity through social interaction (Smith, Gavin, & Sharp, 2015). Briefly, Stage 1 contains identification of conflict between the descriptive norm (‘how the world is)’ and injunctive norm (‘how it should be’); Stage 2 contains communication of perceptions and cognitions about the issues discussed at Stage 1; Stage 3 witnesses the emergence of shared identity centred on increasing consensus regarding injunctive norms (associated with increased use of first‐person plural pronouns and indicators of consensus); Stage 4 is defined by the constituent individuals' shared and aligned perceptions, cognitions and emotions regarding their shared injunctive norm (actions they agree they should take). Localization of stages within the model is based upon linguistic markers. Transcript analysis, breaking down the dyad's discussion into stages according to said model, provided epochs in which DTW within each stage was extracted, permitting analysis of inter‐brain synchrony within and between stages.

### Hypothesis

We hypothesized that stage of social identity formation would moderate the relationship between inter‐brain synchrony and the linguistic markers of social identity (indexed through proportion of first‐person plural pronouns and affiliation words used in a dyad's dialogue (see Figure S1)). Specifically, there would be an interaction between Stage and inter‐brain synchrony, where there would be no relationship between inter‐brain synchrony and the linguistic markers of social identity at Stage 1, because at that stage there was no shared identity, whereas at Stage 3 there would be a positive relationship between inter‐brain synchrony and the linguistic markers of social identity. This is because at Stage 3, a shared social identity has formed between the participants in the dyad, thus there should be greater use of social identity linguistic markers, and greater similarity in EEG signal.

## METHODS

### Participants

Sixty unacquainted participants were recruited from a convenience sample of students and staff within a UK University. This sample size is sizeable compared to similar work in the field. The mean age was 30.9 years of age (*SD* = 13.9 years). All participants were female to control for sex differences in neural activation associated with social processing (Proverbio, 2023; Sato, 2020). All participants identified their sex as female. Fifty‐six participants reported themselves as ‘British residents or a permanent citizen’. All sixty participants reported English being their first language.

### Procedure

Following completion of the online consent procedure, participants were paired with another person with similar scores on the Blatant and Subtle Prejudice Scales to create pairs that were more likely to form a shared social identity, and invited to the Department of Psychology labs. The Blatant and Subtle Prejudice Scales use a four‐point Likert scale to measure blatant (direct and close) and subtle (indirect and distant) prejudice (Pettigrew & Meertens, 1995). We adapted the scale to focus on prejudice towards immigrants by changing the original text ‘West Indians’ with ‘immigrants’. Participants were individually asked to complete the National Adult Reading Test (NART) to assess competency in the English language (Nelson & Willison, 1991). They were also asked to read an article (https://www.independent.co.uk/news/uk/home‐news/syrian‐refugees‐uk‐immigrants‐resettlement‐scheme‐2020‐numbers‐a8044696.html) about the plight of refugees, which would be the topic of discussion within the dyads. Participants were then fitted with separate Emotiv EPOC 14‐channel wireless EEG headsets and seated facing each other across a table. They were then instructed to discuss, for approximately ten minutes, their opinions regarding the key issues of immigration that the UK are facing and some possible actions that people like them, who share similar opinions on immigration, can take to tackle those issues, with no direction given on the nature of any actions that could be agreed upon. Participants were audio‐ and video‐taped during the discussion.

Participants subsequently took part in a control condition where they were separately seated facing the wall and asked to talk about a non‐emotional topic (a table) for one minute. One minute was considered an appropriate time to acquire a continuous EEG signal with movement artefacts and psychological representations related to language production (Valenzi et al., 2014). This allowed capture of EEG waveforms associated with speech production and articulation but without the social element of speaking in a dyad to another person.

### Analysis protocol

We used the INN‐formation model (Table S1) (Smith, Thomas, & McGarty, 2015) to create a coding protocol that described the stages of social identity formation each dyad were at throughout their 10 min interaction. During Stage 1, individuals did not share a social identity on the topic of discussion, whereas in Stage 3, individuals developed a shared social identity premised upon consensus about the ideas raised during the discussion, referred to as an ‘opinion‐based’ social identity (Bliuc et al., 2007; Smith, Gavin, & Sharp, 2015). The coding protocol was applied to transcripts of each dyad's interaction to identify epochs, each of which was associated with a discrete stage within the identity formation process. Epochs were temporal segments of dyads' discussions which provided timing to extract associated segments of the EEG waveform. Dynamic Time Warping (DTW) was used to provide values of inter‐brain synchrony of participants within each dyad for a given epoch. DTW is a Power‐based Connectivity Measure which attempts to align two sequences/signals by iteratively warping the comparison path between their dyadic time‐series data (until an optimal alignment is found) instead of directly comparing the signals based on time (e.g. Euclidean distance) (Miljevic et al., 2023; Sedrati et al., 2023). This gives DTW an advantage over the Euclidean distance, as it facilitates the comparison between signals that are ill‐aligned/out‐of‐phase by stretching/compressing them (Miljevic et al., 2023; Yamauchi et al., 2015) by a specified window of timeframes while aligning them within a wider range (i.e. segmented *Stage*) and before calculating the DTW distance (Sopic et al., 2023). DTW distance value was used to indicate the level of synchrony between dyadic sequences/signals; the larger the DTW distance was, the lesser was the synchronization between the signals (Giorgino, 2009). The most significant advantage which DTW provided for this study was its ability to compare two sequences/signals that vary in speed (Dehzangi et al., 2019). This was particularly important, as the aim of this study was to analyse the synchrony between conversation‐based EEG data where synchronization might not have happened instantly. Another reason for choosing DTW was because it required a single sample for comparison (Steinmeier & Becking, 2020) which was the case in our conversation data in which we had a single segmented signal per each *Stage* and each dyad.

It is important to note that lower DTW scores represented greater similarity between the EEG signals of the people within a dyad during an epoch, so a negative relationship between DTW scores and linguistic markers of social identity represented a positive association between inter‐brain synchrony and social identification.

### EEG acquisition and processing

EEG was acquired using Emotiv EPOC 14‐channel headsets (www.emotiv.com). The wireless, lightweight, headsets were used to promote more of a natural feel to the dyads' interactions compared to static EEG hardware although, to reduce movement artefacts, participants were instructed to keep their heads as still as possible. The Emotiv EPOC headsets provide suitable measurement sensitivity and signal‐noise ratio for use in more naturalistic environments (Badcock et al., 2013; de Lissa et al., 2015; Hairston et al., 2014). Six dyads were removed from analysis due to bad channels pervading the EEG recording.

Sensors are located at AF3, AF4, F3, F4, F7, F8, FC5, FC6, P7, P8, T7, T8, O1, O2 spatially organized via the international 10–20 system. There are two further sensors within each hemisphere that act as reference channels. The sampling rate was 128 Hz.

Emotiv EPOC headsets contain online 5th order since filters to bandpass data between 0.16 and 43 Hz; offline filters were, therefore, not applied to the data. Automatic correction of EOG artefacts was applied in BESA Research 7.0; this procedure uses an internal model of eye artefact topographies and a source montage to separate brain activity from oculomotor activity and remove the latter (Berg & Scherg, 1994) Threshold values for EOG removal were 150 μv for horizontal EOG, and 250 μv for vertical EOG artefacts. The following procedure was developed and carried out to check the original pre‐processing steps and identify, and remove, any further artefacts. A datapoint was considered an outlier and removed from the dataset if the following two conditions were satisfied within an event: Condition 1 – the shortest margin between the values of the selected datapoint and its two immediate neighbours was three‐fold bigger than the margin between the neighbours' values and condition 2 – the shortest margin between the values of the selected datapoint and its two immediate neighbours was bigger than 20% of the interpercentile range. This procedure led to removal of only a negligible quantity of data (a maximum of 1.7% from one participant and, typically, significantly less).

Prior to applying Dynamic Time Warping, EEG data was averaged over all electrodes and the time‐series for extracted epochs within the EEG data were subsequently decomposed into standard EEG frequencies (delta, theta, alpha, beta and gamma). Decomposition of the EEG was accomplished by applying the Butterworth filter (Proakis & Manolakis, 1992) in R using the *butter* function within the *signal* package (Ligges et al., 2021). Signals from the control condition were subtracted from those obtained within the social interaction to remove unwanted signals associated with language and language‐related movement. Regressions between DTW distance and linguistic markers between individual frequency bands did not differ substantially, thus we decided, as we were using an exploratory approach, to collate all frequencies within subsequent analyses.

### Dynamic time warping (DTW)

DTW was conducted in R using the *dtw* package (Giorgino, 2022). Prior to applying DTW, a 1000‐timestep sample was extracted from the baseline signal based on a random starting point, to be used as a non‐conversational reference. This sample was appended to itself until the new combined signal reached the same length as the corresponding ‘conversation’ signal. Then, the baseline signal was subtracted from the ‘conversation’ signal.

Several techniques were implemented to increase the effectiveness of DTW measurements. First, the compared signals within each epoch were *z*‐normalized prior to calculating the DTW scores. The *z*‐normalization transformation shifts and scales the signals such that they will have a mean of *zero* and a standard deviation of *one*; such transformation brings the compared signals to the same scale and makes them commensurate (Rakthanmanon et al., 2013). Secondly, the implementation of Butterworth filter (Proakis & Manolakis, 1992) often resulted in the signals having a large variability at the epochs' endpoints. To remove this distortion in the signals and potential effects on the accuracy of DTW similarity measures, both ends of the epochs were trimmed by 0.1 of the total signal's length from each end prior to calculating DTW scores. Third, the *open*.*end* argument in the *dtw* package was set to TRUE to relax the boundary constraint (Giorgino, 2009) in determining the DTW paths; which helps in minimizing the number of poor matching occurrences, and accordingly, increasing the accuracy of DTW scores. Fourth, a *window* constraint was instituted to reduce the volume of required computation within the DTW matrix; here, Sakoe‐Chiba band was chosen to determine the window's boundaries as it gives the opportunity to each timeframe datum in a signal to be compared with all the data points, within the window, from the corresponding signal. Moreover, Sakoe‐Chiba band is claimed to be superior, in terms of DTW performance, to Itakura Parallelogram constraint method (Geler et al., 2019).

Once the DTW process was completed and the DTW scores were computed, we could then compare the relationship between DTW scores and linguistic indicators of social identity across stages. As DTW distances are sensitive to the sequences' length and amplitude, the DTW scores were normalized by epochs' length and signals' amplitude prior to the linear mixed‐effects analysis; as follows:

$$\text{Normalised}\;\mathrm{DTW}=\frac{\mathrm{DTW}\;\text{score}}{\left(M+N\right)\cdot \left|\mathrm{AU}C_{1}-\mathrm{AU}C_{2}\right|}$$

where *M* is the length of the epoch's first signal (i.e., width of DTW matrix), *N* is the length of the epoch's second signal (i.e., height of DTW matrix), AUC_1_ is the Area Under the Curve (AUC) of the epoch's first signal, AUC_2_ is the Area Under the Curve (AUC) of the epoch's second signal.

### Categorization of the stages of social identity formation

By qualitatively coding the transcripts of the dyads' discussions, we were able to manually identify the stage of social identity formation that dyads were in at any point during their discussion. The purpose of this was to identify relevant epochs of time within the EEG signal.

The coding of stages of social identity formation for each dyad was carried out by one trained coder with the coding checked and corroborated by the first author. The coder was trained by the lead author of the conceptual model (L.G.E. Smith). Codes were checked and corroborated by the first author, independent of Smith. The stages of the model are outlined within the introduction. Table S2 shows the coding protocol for these categories in detail. There was an insufficient number of Stage 4 epochs for robust analysis (five in total), thus we focused on Stage 1–3 epochs (Stage 3 was the most pertinent, as during this stage a shared identity emerged). Epochs were collated by stage, and we controlled for the duration of epochs in our analyses.

### Measurement of linguistic markers

To investigate how inter‐brain synchrony (DTW scores) was associated with social identification, we created two quantitative variables that provided metrics for linguistic markers of social identification (first person plural pronouns, e.g., ‘we’, ‘us’, ‘our’; and affiliation talk, e.g., ‘ally’, ‘friend’). This was performed by running Linguistic Inquiry and Word Count (LIWC) 2015 software (Tausczik & Pennebaker, 2010) on each epoch extracted from each dyad's interaction. This provided proportions of the total words used by each dyad that appeared in the *first‐personal plural pronouns* and *affiliation* LIWC dictionaries. These variables provided metrics of social identity language that we could model with DTW scores (the indicator of neural synchrony). As the distribution of the proportion of affiliation words and first‐person plural pronouns tended towards excess positive skew and excess kurtosis (Tables S3 and S4), we log10 transformed the scores, which normalized the distribution of the data. LIWC also provides a variable for the total word count of the dialogue within each epoch, which we included as a control variable.

### Analytic strategy

First, we calculated mean usage of first‐person plural pronouns and affiliation words across stages. The aim of this analysis was to investigate whether the linguistic markers of social identity formation varied as expected across the stages of identity formation, and to validate the manual coding of the stages of identity formation. The identity‐norm nexus model suggests that there should be a higher proportion of social identity linguistic markers in later stages of identity formation.

Second, to test the hypothesis, we conducted two linear mixed‐effects models (LMER) in *R* using the *lme4* package (Bates et al., 2015). The models included random intercepts at the dyad level to account for the interdependence of scores within dyads. The first LMER predicted (log‐transformed) first person plural pronouns from two dummy variables representing the stages of social identity formation, and DTW scores, controlling for epoch word count and duration, and included the two‐way interactions between each dummy variable and DTW scores. The second model included the same predictors, but the dependent variable was affiliation talk. We then conducted a simple slopes analysis to decompose significant interactions.

## RESULTS

Table 1 shows the means for the linguistic variables across stages. Repeated‐measures ANOVA indicated that the means differed significantly between groups as indicated in Table 1.

**TABLE 1:** Mean values and standard deviations of (non‐transformed) frequency of affiliation and first‐person plural pronouns within each stage of social identity formation.

	Stage of social identity formation		
	1	2	3
	Mean	Mean	Mean
Mean affiliation words	2.07_a_ (2.48)	2.46_b_ (2.84)	2.99_b_ (3.31)
Mean first person plural pronouns	1.21_a_ (2.17)	1.76_b_ (2.36)	1.27_ab_ (2.26)
Mean duration of epochs (s)	29.75_a_ (22.49)	15.46_b_ (14.31)	21.16_b_ (15.74)
Mean word count within epochs	86.99_a_ (61.10)	48.42_b_ (39.29)	66.18_b_ (48.60)
Mean DTW distance	0.068_a_ (0.052)	0.11_b_ (0.087)	0.096_c_ (0.073)

*Note*: Standard deviations are shown within parentheses. Means on rows with different subscripts are significantly different at *p* < .05. Mean DTW distance are transformed values predicted by the regression model.

Affiliation words and first‐person plural pronouns were calculated as proportions of total word count per dyad and averaged across dyads for each stage. Mean (and *SD*) DTW distance over EEG spatial locations used for analysis are found in Tables S5–S9.

The results of the linear mixed‐effects modelling are reported in Tables 2 and 3. For the first model, which predicted first‐person plural pronouns (Table 2), there was a main effect of DTW distance on first person plural pronouns, *γ* = −.00007, *t*(374) = −2.18, *p* < .05. This is evidence that DTW values, which indicate greater synchrony in EEG waveforms between the individuals within a dyad, were negatively associated with the linguistic markers of a shared social identity. There was also a significant negative main effect for epoch word count, but no other significant main effects (Table 2).

**TABLE 2:** LMER results predicting first person plural pronouns from DTW distance and stage (epochs, *n* = 374; dyads, *N* = 24).

Predictor	First person plural pronouns (log10 transformed)					
	*γ*	*SE*	*t*	*F*	*σ* ^2^	*SD*
Random effects						
Dyad (Intercept)					.02	0.14
Residual					.06	0.25
Fixed effects						
Intercept	0.73	0.05	13.50			
Epoch word count	−0.004	0.001	−8.30*	86.16		
Epoch duration	0.003	0.002	1.53	2.79		
DTW scores	−0.00007	0.0003	−2.18*	110.16		
Dummy representing Stage 3 (vs. 1)	−0.09	0.05	−1.07	7.23		
Dummy representing Stage 2 (vs. 1)	−0.08	0.07	−1.08	5.34		
Interaction between dummy representing Stage 3 (vs. 1) and DTW scores	0.00007	0.00003	2.49*	3.81		
Interaction between dummy representing Stage 2 (vs. 1) and DTW scores	0.00008	0.00005	1.70	2.89		

Abbreviation: DTW, Dynamic Time Warping.

* *p* < .05. A value of the *t* statistic greater or equal to 1.96 indicates a significant effect.

**TABLE 3:** LMER results predicting affiliation words from DTW distance and stage (epochs, *n* = 374; dyads, *N* = 24).

Predictor	Affiliation talk (log10 transformed)					
	*γ*	*SE*	*t*	*F*	*σ* ^2^	*SD*
Random effects						
Dyad (Intercept)					.01	0.12
Residual					.07	0.26
Fixed effects						
Intercept	0.80	0.04	17.03			
Epoch word count	−0.003	0.0005	−5.89*	49.28		
Epoch duration	0.002	0.002	1.07	1.27		
DTW scores	−0.00003	0.00003	−0.99	76.02		
Dummy representing Stage 3 (vs. 1)	−0.12	0.05	−2.61*	4.45		
Dummy representing Stage 2 (vs. 1)	−0.05	0.06	−0.78	2.29		
Interaction between dummy representing Stage 3 (vs. 1) and DTW scores	0.00003	0.00002	1.23	2.25		
Interaction between dummy representing Stage 2 (vs. 1) and DTW scores	−0.00001	0.0004	−0.30	0.09		

Abbreviation: DTW, Dynamic Time Warping.

* *p* < .05. A value of the *t* statistic greater or equal to 1.96 indicates a significant effect.

We found a significant interaction between the dummy representing Stage 3 (vs. 1) and DTW scores (Table 2). As stated in the previous paragraph, at Stage 3 (compared to 1) there was a significant negative relationship between DTW scores and first‐person plural pronouns. However, at Stage 1 (vs. 3), DTW scores did not significantly predict first person plural pronouns, *γ* = .00008, *t*(374) = 1.46, *p* > .05. Therefore, supporting our hypothesis, DTW scores were only related to use of language associated with social identity during Stage 3 epochs.

In the second LMER model (Table 3), there was no main effect of DTW distance on affiliation words, *γ* − .00003, *t*(374) = −0.99, *p >* .05, but there was a significant difference between Stage 3 epochs and Stage 1 epochs on use of affiliation words, *γ* = −.12, *t*(374) = −2.61, *p* < 05, with more used at Stage 3. However, the interaction between Stage 3 (vs. 1) and DTW scores was not significant, *γ* = .00003, *t*(374) = 1.23, *p* > 05, suggesting that the relationship between DTW scores and use of affiliation words did not differ across stages.

## DISCUSSION

This study took an empirically and theoretically grounded approach to investigating the relationship between social identity formation and neurophysiological activity between dyads within a naturalistic social interaction. We found that greater inter‐brain synchrony between dyads during the latter stage of identity formation was associated with higher use of first‐person plural pronouns, which suggested the emergence of a shared identity at both a behavioural and a neural level. Importantly, the findings showed that there was greater neural synchrony between interacting individuals during the emergence of a shared identity versus other stages of their social interaction.

The results have shown that inter‐brain synchrony is related to the establishment of a social identity, whilst controlling for other factors that may affect this relationship, such as the amount and duration of dialogue. This aligned with previous work showing that greater levels of synchronization in EEG oscillatory waveforms during social interaction was associated with participant reports of connectedness with others (Dikker et al., 2017; Hu et al., 2018; Valencia & Froese, 2020). Shared social identification is crucial to the formation of social connectedness (Haslam et al., 2022). Our results have shown a potential link between inter‐brain synchrony, social psychological connectedness, and the formation of social identity. Furthermore, we extend previous work by mapping the emergence of a shared social identity from an initial state where members of the dyad have never met and are not affiliated with each other, thus our results have applications in understanding inter‐brain synchrony in naturalistic conversations where individuals are initially naïve to each other. Separately, inter‐brain synchrony has been associated with increases in team effectiveness (Likens et al., 2014; Réveillé et al., 2024; Stevens & Galloway, 2016; Szymanski et al., 2017). Our method and findings may be useful in elucidating the relationship between inter‐brain synchrony, social identity and interaction, providing scope to be able to predict the emergence of shared identity following further refinement of the method.

Second, we validate the relationship between inter‐brain synchrony and social pronoun usage. Our work illuminates new research opportunities within social psychology. Developing such a method is of value to both academics and non‐academics. It permits social psychologists new measures to rapidly detect social identity formation, a core foundation of ingroup formation and the behaviours that come about from its establishment. Practical applications include monitoring of social interaction and a method to measure whether psychological interventions designed to facilitate social identity formation do so at the time of formation, rather than a post‐hoc measurement at a time point distant to the group interaction.

Our method captures the dynamic relationship between inter‐brain synchrony and social identity formation. In practice, measurement of IBS could be used to infer the formation of social identification within groups. For example, in co‐operative scenarios, or negotiations, IBS could be used to signal whether group members are in a state where consensualization is more possible due to the forming of social identification within the group. Applications designed to promote social identification, e.g. virtual environments, could also measure IBS as a proxy for formation of social identification, permitting testing of different strategies.

Although the results were broadly consistent with our hypotheses, the interaction between Stage and DTW scores was significant only for usage of first‐person plural pronouns and not for affiliation words, and the analysis of the usage of first‐person plural pronouns and affiliation words did not show the same pattern of results across conditions. This could be due to the differences between the words within the first‐person plural and affiliation categories as defined by LIWC. First‐person plural pronouns would be those that typically denote existence of connectedness within the conversing dyads, e.g., ‘we’, ‘us’, ‘our’. Affiliation words also denote connectedness, but these words could easily be used to denote connectedness of others as well as the conversing dyads, e.g., ‘ally’, ‘friend’, ‘mate’. In other words, the first‐person plural pronouns LIWC dictionary was more directly able to capture shared identification than the affiliation LIWC dictionary, however, we chose to use both because they both can indicate shared identity.

One may, conceivably, expect IBS and the linguistic markers of social identity to increase linearly with stage progression, however we did not find full support for this idea. Within the conceptual model, Stage 1 is defined by a description of the issues with no reference to pronoun usage, whereas Stage 2 is defined by elaboration of reaction towards the issues identified with reference to usage of first‐person singular pronouns. Only Stage 3, the emergence of shared identity centred on increasing consensus, is defined by increased usage of first‐person plural pronouns. Thus, IBS associated with greater usage of first‐person plural pronouns should only be significantly higher in Stage 3, as seen in the findings.

The findings move beyond IBS as generally representing a connection of some sort within interacting individuals to grounding it within established theory within social psychology. IBS research has been criticized as lacking in theory (Holroyd, 2022). Our research meshes established theory within social psychology with IBS. Application of social identity theory may help explain what IBS is representing in a broad range of the observations within the literature, i.e. that IBS represents the building of social identification. Furthermore, alignment with the INN‐formation model allows clearer insight into when IBS should be expected to be observed, and when it should not. Future research should scrutinize the application of this theory to existing and new findings. Further research could also focus on contextual effects of the relationship between inter‐brain synchrony and social identity, for example formal versus informal relationships. Cross‐cultural studies could examine how the relationship holds across different cultures, which may benefit from inter‐disciplinary collaboration between psychologists, sociologists, anthropologists and linguists.

In conclusion, we have shown that EEG can be used in naturalistic interaction to identify the emergence of shared social identity, a crucial process in the formation of social groups. We show that inter‐brain synchrony occurs not simply by engaging in a similar task, but within deeper‐level psychological processes that underlie our social behaviour. The work has applications within, and outside of, psychological science in any area where the formation of social psychological groups between interacting individuals is of interest.

## AUTHOR CONTRIBUTIONS

**Neal S. Hinvest:** Conceptualization; methodology; data curation; validation; formal analysis; supervision; funding acquisition; visualization; project administration; writing – original draft; writing – review and editing. **Chris Ashwin:** Conceptualization; methodology; visualization; funding acquisition; writing – review and editing. **Muhammad Hijazy:** Data curation; formal analysis; visualization; writing – review and editing. **Felix Carter:** Data curation; formal analysis; investigation. **Chiara Scarampi:** Data curation; formal analysis; investigation. **George Stothart:** Conceptualization; methodology; formal analysis; visualization; supervision; writing – review and editing. **Laura G. E. Smith:** Conceptualization; data curation; methodology; visualization; funding acquisition; writing – review and editing.

## conflict of interest STATEMENT

The authors declare that they have no conflicts of interest.

## Supporting information


Appendix S1.

## Data Availability

All data can be accessed via the Open Science Framework at https://osf.io/6j95r/?view_only=7f77747ea53f42389898cfbbaa6481a6.
