# Supplementary material for: Inter‐brain synchrony is associated with greater shared identity within naturalistic conversational pairs
Source: Br J Psychol. 2024 Oct 26;116(1):170–82. doi: 10.1111/bjop.12743 (PMC11724682; doi:10.1111/bjop.12743)
Supplement: Supplementary file 1 — Appendix S1. [file BJOP-116-170-s001.docx]

**Supplementary Material: Inter-brain synchrony is associated with greater shared identity within naturalistic conversational pairs**

**Hypothesis**

We hypothesised that the stage of social identity formation (Table S1) will moderate the relationship between DTW scores and first-person plural/affiliation LIWC scores (Figure S1). Specifically, at stage 1, we hypothesised that there would be no relationship between DTW and first-person plural/affiliation LIWC scores (H1a), and at Stage 3 there would be a negative relationship between DTW scores and first personal plural/affiliation LIWC scores (H1b).

**Figure S1. Hypothesised Model.**In the figure, first-person plural words are referred to as “we” words for conciseness.


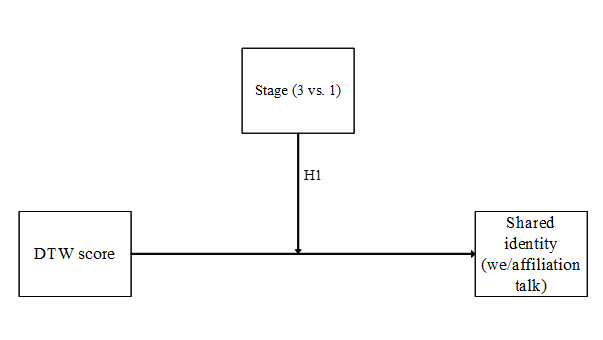


**Table S1. The stages of shared social identity formation as manifested linguistically during social interaction.**

| ***Identity Formation Stage*** | ***Linguistic Markers*** |
| --- | --- |
| Stage 1: Identification of conflict between descriptive norm (‘how the world is)’ and injunctive norm (‘how it should be’) | No predictive linguistic markers. |
| Stage 2: Communication of perceptions and cognitions about (1) | Words indicating sense of grievance, anger, outrage, injustice; Greater use of first person singular pronouns (e.g., “I”, “you”) than first person plural pronouns (e.g., “we”, “us”, “our”) in relation to opinions on (1); some disagreement about injunctive norm (e.g., “no”, “I disagree”). |
|  |  |
| Stage 3: Emergence of shared identity centred on increasing consensus on ideas about injunctive norm | Increased agreement about injunctive norm (indicators of consensus, e.g., “absolutely”, “ that’s right”).  Increased use of first-person plural, pronouns (e.g.  “we”, “us”, “our”) relative to first-person singular pronouns (“I”, “you”); |
| Stage 4: Establishment of shared social identity premised on consensus about injunctive norm | Use of first-person plural pronouns (e.g.  “we”, “us”, “our”) in reference to consensual ideas about injunctive norm; marked presence of agreement (indicators of consensus, e.g., “absolutely”, “that’s right”) in relation to key ideas about shared injunctive norm coupled with a relative absence of disagreement |

**Table S2: Coding framework based upon the identity-norm nexus model (Smith et al, 2015).**

| **Stage** | **Code** | **Definition of code/examples** |
| --- | --- | --- |
| Description of issues (rather than a reaction to them) - list of issues | 1 | DESCRIPTION OF ISSUE(S).    Ppts recognise/list issues re. immigration that the UK is facing compared to what ‘should’ be happening.    Subcode - 1a - issues listed in the article; 1b - new issues not in article.  Subcode - 1aa = positive towards immigrants  1ab = negative towards immigrants  1ba = positive towards immigrants  1bb = negative towards immigrants    Subcode = add ‘c’ to above codes to indicate consensus/agreement/validation |
| An individual experiences a normative  conflict and communicates of perceptions and  cognitions | 2 | ELABORATION OF REACTION TOWARDS ISSUE(S).  An individual perceives an unacceptable violation of his/her personal ideals by a descriptive social norm manifesting in an existing group.  The individual develops ideas about the change(s) she or he wants to see in the world (personal ideas about the injunctive norm).    Look for words like ‘should’ and ‘ought’. Evidence of awareness of a problem that needs to be addressed/something needs to change.    Talking about emotional / moral response to the issue above. Elaboration on their own reaction and understanding of the issue.    Subcode - 2a - issues listed in the article; 2b - new issues not in article.  Subcode - 2aa = positive towards immigrants  2ab = negative towards immigrants  2ba = positive towards immigrants  2bb = negative towards immigrants    Subcode = add ‘c’ to above codes to indicate consensus/agreement/validation |
| Communicating individuals develop  ideas about the change(s) they want  to see in the world (ideas for shared  injunctive norm).    Talking about actions | 3 | EMERGENCE OF SOCIAL IDENTITY    Description of actions that can tackle the issue(s) above.    Subcodes - 3a - agreement on action ideas (nodding/’yes’, ‘I agree’, etc).  3b - social validation - ‘that’s a good idea’; ‘that would work’; |
| Formation of identity-norm nexus (shared social identity)    Agreeing to take those actions together | 4 | ESTABLISHED SOCIAL IDENTITY LEADING TO JOINT ACTION    Those communicating individuals perceive their perceptions, cognitions, and emotions are validated/shared (or not validated/shared) by each other to some extent. Communicators with shared views define themselves as members of a distinct social category (commonly  self-categorize). The category is defined by the constituent individuals’  shared and aligned perceptions, cognitions, and emotions about the injunctive norm (identity-norm nexus; INN); Shared cognition about  reality emerges.    Evidence of shared identity (shared self-definition) often found in pronoun use:    4a = overall change from first person singular pronouns to first person plural pronouns  4b = use of first person plural pronouns (e.g., ‘we’, ‘us’, ‘our’) in relation to dyad  4c = evidence of shared opinion on issue(s) and action(s), ‘we should do that’ - agreeing to take shared action |
| Sociopolitical action | 5 | Assessed by post-discussion questionnaire |
| Expression of sharedness/bonding (about anything) | 6 | Moment of bonding (compare to first minute of discussion). With expression of energy/positive emotion. |
| Formation of shared identity vs. not (at the transcript/dyad level) | 7 | Either 7a = did reach a shared sense of identity (on any issue, including being participants) or 7b = did not reach a shared sense of identity |

**Table S3. Skew and Kurtosis of number of first-person plural category words split by stage of the social identification model.**

|  | Stage of the social identification model | | |
| --- | --- | --- | --- |
|  | Stage 1 | Stage 2 | Stage 3 |
| Skew | 2.75 | 1.26 | 2.93 |
| Kurtosis | 12.25 | 3.97 | 14.13 |

**Table S4. Skew and Kurtosis of number of affiliation category words split by stage of the social identification model.**

|  | Stage of the social identification model | | |
| --- | --- | --- | --- |
|  | Stage 1 | Stage 2 | Stage 3 |
| Skew | 1.69 | 1.08 | 1.30 |
| Kurtosis | 6.52 | 3.43 | 4.35 |

**Table S5. Mean (and *SD*) DTW distance over all electrodes split by dyad and frequency bandwidth**

| Group | Delta mean | Delta *SD* | Theta mean | Theta *SD* | Alpha mean | Alpha *SD* | Beta mean | Beta *SD* | Gamma mean | Gamma *SD* |
| --- | --- | --- | --- | --- | --- | --- | --- | --- | --- | --- |
| 1 | 0.2727 | 0.0204 | 0.0059 | 0.0004 | 0.0047 | 0.0007 | 0.0085 | 0.0005 | 0.0067 | 0.0002 |
| 3 | 0.3539 | 0.1597 | 0.0059 | 0.0018 | 0.0051 | 0.0020 | 0.0070 | 0.0031 | 0.0045 | 0.0022 |
| 6 | 0.4857 | 0.1355 | 0.0060 | 0.0012 | 0.0063 | 0.0012 | 0.0194 | 0.0060 | 0.0174 | 0.0050 |
| 7 | 0.0994 | 0.0545 | 0.0243 | 0.0056 | 0.0093 | 0.0007 | 0.0048 | 0.0008 | 0.0082 | 0.0009 |
| 8 | 0.1323 | 0.0275 | 0.0082 | 0.0045 | 0.0060 | 0.0030 | 0.0042 | 0.0014 | 0.0027 | 0.0008 |
| 9 | 0.2405 | 0.0467 | 0.0090 | 0.0023 | 0.0047 | 0.0010 | 0.0053 | 0.0004 | 0.0040 | 0.0005 |
| 10 | 0.1182 | 0.0647 | 0.0098 | 0.0031 | 0.0084 | 0.0026 | 0.0074 | 0.0021 | 0.0112 | 0.0022 |
| 11 | 0.5444 | 0.2365 | 0.0064 | 0.0034 | 0.0083 | 0.0030 | 0.0221 | 0.0034 | 0.0193 | 0.0044 |
| 12 | 0.1799 | 0.0412 | 0.0049 | 0.0008 | 0.0037 | 0.0003 | 0.0070 | 0.0009 | 0.0063 | 0.0008 |
| 13 | 0.2272 | 0.0984 | 0.0039 | 0.0017 | 0.0047 | 0.0020 | 0.0133 | 0.0072 | 0.0119 | 0.0074 |
| 15 | 0.0413 | <0.0001 | 0.0016 | <0.0001 | 0.0010 | 0.0000 | 0.0015 | <0.0001 | 0.0008 | <0.0001 |
| 16 | 0.2987 | 0.1038 | 0.0071 | 0.0026 | 0.0084 | 0.0011 | 0.0192 | 0.0027 | 0.0166 | 0.0021 |
| 17 | 0.2670 | 0.2202 | 0.0077 | 0.0034 | 0.0064 | 0.0027 | 0.0099 | 0.0040 | 0.0077 | 0.0028 |
| 18 | 0.2735 | 0.1512 | 0.0150 | 0.0033 | 0.0171 | 0.0094 | 0.0122 | 0.0070 | 0.0068 | 0.0041 |
| 20 | 0.0497 | 0.0054 | 0.0067 | 0.0039 | 0.0053 | 0.0032 | 0.0034 | 0.0018 | 0.0017 | 0.0007 |
| 21 | 0.4279 | 0.3153 | 0.0088 | 0.0034 | 0.0079 | 0.0015 | 0.0109 | 0.0045 | 0.0070 | 0.0034 |
| 22 | 0.3036 | 0.2085 | 0.0039 | 0.0021 | 0.0041 | 0.0020 | 0.0106 | 0.0058 | 0.0097 | 0.0065 |
| 24 | 0.1940 | 0.0843 | 0.0071 | 0.0018 | 0.0037 | 0.0013 | 0.0091 | 0.0018 | 0.0076 | 0.0017 |
| 25 | 0.1794 | 0.0472 | 0.0341 | 0.0086 | 0.0210 | 0.0048 | 0.0077 | 0.0021 | 0.0040 | 0.0010 |
| 26 | 0.3142 | 0.1527 | 0.0059 | 0.0017 | 0.0056 | 0.0010 | 0.0111 | 0.0017 | 0.0101 | 0.0027 |
| 27 | 0.0786 | 0.0320 | 0.0129 | 0.0031 | 0.0064 | 0.0019 | 0.0035 | 0.0008 | 0.0031 | 0.0006 |
| 28 | 0.3107 | 0.2002 | 0.0042 | 0.0010 | 0.0031 | 0.0012 | 0.0059 | 0.0029 | 0.0044 | 0.0020 |
| 29 | 0.2173 | 0.0751 | 0.0076 | 0.0015 | 0.0078 | 0.0024 | 0.0213 | 0.0074 | 0.0216 | 0.0072 |
| 30 | 0.0991 | 0.0185 | 0.0061 | 0.0025 | 0.0054 | 0.0027 | 0.0042 | 0.0017 | 0.0023 | 0.0009 |

**Table S6. Mean (and *SD*) DTW distance over anterior region electrodes (AF3, AF4, F3, F4,) split by dyad and frequency bandwidth.**

| Group | Delta mean | Delta *SD* | Theta mean | Theta *SD* | Alpha mean | Alpha *SD* | Beta mean | Beta *SD* | Gamma mean | Gamma *SD* |
| --- | --- | --- | --- | --- | --- | --- | --- | --- | --- | --- |
| 1 | 0.3518 | 0.0556 | 0.0082 | 0.0021 | 0.0073 | 0.0027 | 0.0106 | 0.0018 | 0.0082 | 0.0011 |
| 3 | 0.2729 | 0.1268 | 0.0063 | 0.0023 | 0.0058 | 0.0023 | 0.0076 | 0.0033 | 0.0050 | 0.0023 |
| 6 | 0.3704 | 0.1015 | 0.0081 | 0.0009 | 0.0074 | 0.0013 | 0.0215 | 0.0053 | 0.0195 | 0.0048 |
| 7 | 0.0982 | 0.0554 | 0.0242 | 0.0059 | 0.0093 | 0.0008 | 0.0051 | 0.0009 | 0.0085 | 0.0010 |
| 8 | 0.1290 | 0.0325 | 0.0071 | 0.0041 | 0.0054 | 0.0021 | 0.0051 | 0.0016 | 0.0037 | 0.0012 |
| 9 | 0.1308 | 0.0540 | 0.0078 | 0.0018 | 0.0039 | 0.0008 | 0.0035 | 0.0003 | 0.0024 | 0.0002 |
| 10 | 0.1296 | 0.0775 | 0.0094 | 0.0030 | 0.0081 | 0.0025 | 0.0073 | 0.0021 | 0.0106 | 0.0023 |
| 11 | 0.3165 | 0.1412 | 0.0082 | 0.0033 | 0.0095 | 0.0028 | 0.0222 | 0.0040 | 0.0193 | 0.0043 |
| 12 | 0.1836 | 0.0492 | 0.0058 | 0.0015 | 0.0045 | 0.0011 | 0.0094 | 0.0027 | 0.0088 | 0.0022 |
| 13 | 0.1621 | 0.0538 | 0.0049 | 0.0019 | 0.0045 | 0.0017 | 0.0106 | 0.0054 | 0.0095 | 0.0054 |
| 15 | 0.0380 | 0.0000 | 0.0016 | <0.0001 | 0.0010 | <0.0001 | 0.0009 | <0.0001 | 0.0006 | <0.0001 |
| 16 | 0.1271 | 0.0302 | 0.0058 | 0.0017 | 0.0054 | 0.0017 | 0.0118 | 0.0052 | 0.0115 | 0.0047 |
| 17 | 0.2229 | 0.1634 | 0.0094 | 0.0056 | 0.0070 | 0.0033 | 0.0106 | 0.0045 | 0.0079 | 0.0027 |
| 18 | 0.1163 | 0.0126 | 0.0109 | 0.0095 | 0.0082 | 0.0034 | 0.0042 | 0.0018 | 0.0024 | 0.0009 |
| 20 | 0.0673 | 0.0062 | 0.0108 | 0.0038 | 0.0080 | 0.0028 | 0.0051 | 0.0014 | 0.0024 | 0.0005 |
| 21 | 0.2983 | 0.2364 | 0.0110 | 0.0029 | 0.0084 | 0.0013 | 0.0110 | 0.0039 | 0.0072 | 0.0032 |
| 22 | 0.1846 | 0.0940 | 0.0060 | 0.0031 | 0.0055 | 0.0029 | 0.0130 | 0.0090 | 0.0130 | 0.0113 |
| 24 | 0.2343 | 0.1112 | 0.0075 | 0.0020 | 0.0052 | 0.0016 | 0.0124 | 0.0023 | 0.0102 | 0.0017 |
| 25 | 0.2589 | 0.0323 | 0.0146 | 0.0028 | 0.0076 | 0.0011 | 0.0072 | 0.0006 | 0.0049 | 0.0001 |
| 26 | 0.2277 | 0.1255 | 0.0067 | 0.0023 | 0.0057 | 0.0012 | 0.0109 | 0.0019 | 0.0098 | 0.0024 |
| 27 | 0.0759 | 0.0329 | 0.0118 | 0.0029 | 0.0060 | 0.0014 | 0.0034 | 0.0007 | 0.0029 | 0.0005 |
| 28 | 0.1890 | 0.1037 | 0.0059 | 0.0020 | 0.0041 | 0.0017 | 0.0069 | 0.0033 | 0.0044 | 0.0018 |
| 29 | 0.1315 | 0.0397 | 0.0091 | 0.0015 | 0.0080 | 0.0023 | 0.0197 | 0.0064 | 0.0199 | 0.0063 |
| 30 | 0.1008 | 0.0209 | 0.0090 | 0.0027 | 0.0050 | 0.0019 | 0.0044 | 0.0013 | 0.0023 | 0.0008 |

**Table S7. Mean (and *SD*) DTW distance over posterior region electrodes (P7, P8, O1, O2) split by dyad and frequency bandwidth.**

| Group | Delta mean | Delta *SD* | Theta mean | Theta *SD* | Alpha mean | Alpha *SD* | Beta mean | Beta *SD* | Gamma mean | Gamma *SD* |
| --- | --- | --- | --- | --- | --- | --- | --- | --- | --- | --- |
| 1 | 0.3964 | 0.0705 | 0.0061 | 0.0021 | 0.0058 | 0.0013 | 0.0133 | 0.0038 | 0.0107 | 0.0020 |
| 3 | 0.2749 | 0.1308 | 0.0059 | 0.0021 | 0.0061 | 0.0025 | 0.0072 | 0.0033 | 0.0049 | 0.0023 |
| 6 | 0.8574 | 0.0855 | 0.0050 | 0.0016 | 0.0080 | 0.0015 | 0.0241 | 0.0043 | 0.0219 | 0.0040 |
| 7 | 0.1020 | 0.0522 | 0.0253 | 0.0055 | 0.0098 | 0.0004 | 0.0050 | 0.0007 | 0.0085 | 0.0009 |
| 8 | 0.0937 | 0.0152 | 0.0090 | 0.0041 | 0.0064 | 0.0030 | 0.0039 | 0.0014 | 0.0020 | 0.0006 |
| 9 | 1.0173 | 0.0559 | 0.0087 | 0.0018 | 0.0084 | 0.0009 | 0.0164 | 0.0015 | 0.0122 | 0.0024 |
| 10 | 0.1180 | 0.0722 | 0.0112 | 0.0030 | 0.0088 | 0.0023 | 0.0080 | 0.0020 | 0.0112 | 0.0020 |
| 11 | 0.6640 | 0.3338 | 0.0040 | 0.0023 | 0.0077 | 0.0037 | 0.0187 | 0.0078 | 0.0160 | 0.0071 |
| 12 | 0.2133 | 0.0802 | 0.0048 | 0.0017 | 0.0047 | 0.0012 | 0.0091 | 0.0027 | 0.0080 | 0.0026 |
| 13 | 0.2257 | 0.0972 | 0.0026 | 0.0011 | 0.0046 | 0.0021 | 0.0116 | 0.0068 | 0.0103 | 0.0070 |
| 15 | 0.0499 | <0.0001 | 0.0019 | <0.0001 | 0.0016 | <0.0001 | 0.0058 | 0.0000 | 0.0021 | <0.0001 |
| 16 | 0.4228 | 0.0770 | 0.0051 | 0.0016 | 0.0080 | 0.0018 | 0.0215 | 0.0040 | 0.0169 | 0.0039 |
| 17 | 0.3358 | 0.2692 | 0.0046 | 0.0007 | 0.0053 | 0.0023 | 0.0083 | 0.0034 | 0.0070 | 0.0029 |
| 18 | 0.1765 | 0.0560 | 0.0042 | 0.0020 | 0.0026 | 0.0009 | 0.0025 | 0.0013 | 0.0017 | 0.0010 |
| 20 | 0.0583 | 0.0178 | 0.0052 | 0.0028 | 0.0046 | 0.0026 | 0.0032 | 0.0016 | 0.0018 | 0.0006 |
| 21 | 0.4557 | 0.3032 | 0.0091 | 0.0032 | 0.0099 | 0.0013 | 0.0145 | 0.0066 | 0.0089 | 0.0041 |
| 22 | 0.3954 | 0.2127 | 0.0029 | 0.0015 | 0.0049 | 0.0026 | 0.0141 | 0.0092 | 0.0141 | 0.0112 |
| 24 | 0.1712 | 0.0573 | 0.0065 | 0.0022 | 0.0028 | 0.0004 | 0.0054 | 0.0015 | 0.0045 | 0.0014 |
| 25 | 0.5272 | 0.0725 | 0.0037 | 0.0003 | 0.0045 | 0.0003 | 0.0085 | 0.0002 | 0.0060 | 0.0009 |
| 26 | 0.1961 | 0.0459 | 0.0100 | 0.0055 | 0.0077 | 0.0027 | 0.0095 | 0.0020 | 0.0078 | 0.0024 |
| 27 | 0.0878 | 0.0304 | 0.0131 | 0.0031 | 0.0065 | 0.0016 | 0.0035 | 0.0008 | 0.0031 | 0.0006 |
| 28 | 0.2897 | 0.1628 | 0.0032 | 0.0005 | 0.0030 | 0.0010 | 0.0057 | 0.0026 | 0.0042 | 0.0017 |
| 29 | 0.3436 | 0.1196 | 0.0053 | 0.0014 | 0.0084 | 0.0030 | 0.0231 | 0.0095 | 0.0231 | 0.0094 |
| 30 | 0.1116 | 0.0399 | 0.0028 | 0.0015 | 0.0050 | 0.0030 | 0.0041 | 0.0018 | 0.0023 | 0.0010 |

**Table S8. Mean (and *SD*) DTW distance over left region electrodes (F7, FC5, T7) split by dyad and frequency bandwidth.**

| Group | Delta mean | Delta *SD* | Theta mean | Theta *SD* | Alpha mean | Alpha *SD* | Beta mean | Beta *SD* | Gamma mean | Gamma *SD* |
| --- | --- | --- | --- | --- | --- | --- | --- | --- | --- | --- |
| 1 | 0.1980 | 0.1181 | 0.0102 | 0.0051 | 0.0054 | 0.0027 | 0.0077 | 0.0035 | 0.0058 | 0.0024 |
| 3 | 0.3847 | 0.1787 | 0.0075 | 0.0025 | 0.0048 | 0.0017 | 0.0059 | 0.0025 | 0.0043 | 0.0020 |
| 6 | 0.1533 | 0.0474 | 0.0047 | 0.0013 | 0.0029 | 0.0012 | 0.0094 | 0.0059 | 0.0086 | 0.0053 |
| 7 | 0.1443 | 0.0331 | 0.0110 | 0.0017 | 0.0075 | 0.0036 | 0.0089 | 0.0023 | 0.0145 | 0.0028 |
| 8 | 0.1492 | 0.0239 | 0.0088 | 0.0044 | 0.0054 | 0.0024 | 0.0041 | 0.0014 | 0.0029 | 0.0009 |
| 9 | 0.2919 | 0.0644 | 0.0092 | 0.0007 | 0.0055 | 0.0010 | 0.0055 | 0.0014 | 0.0045 | 0.0014 |
| 10 | 0.1170 | 0.0757 | 0.0081 | 0.0026 | 0.0077 | 0.0022 | 0.0067 | 0.0018 | 0.0111 | 0.0019 |
| 11 | 0.3981 | 0.0365 | 0.0070 | 0.0042 | 0.0085 | 0.0035 | 0.0239 | 0.0073 | 0.0206 | 0.0069 |
| 12 | 0.0930 | 0.0295 | 0.0060 | 0.0020 | 0.0037 | 0.0012 | 0.0057 | 0.0015 | 0.0050 | 0.0014 |
| 13 | 0.1423 | 0.0400 | 0.0054 | 0.0028 | 0.0040 | 0.0014 | 0.0100 | 0.0047 | 0.0088 | 0.0047 |
| 15 | 0.0388 | <0.0001 | 0.0015 | <0.0001 | 0.0009 | <0.0001 | 0.0008 | <0.0001 | 0.0006 | <0.0001 |
| 16 | 0.1231 | 0.0626 | 0.0063 | 0.0031 | 0.0067 | 0.0038 | 0.0114 | 0.0063 | 0.0093 | 0.0047 |
| 17 | 0.1738 | 0.1775 | 0.0071 | 0.0029 | 0.0062 | 0.0027 | 0.0097 | 0.0043 | 0.0078 | 0.0035 |
| 18 | 0.1351 | 0.0353 | 0.0041 | 0.0011 | 0.0019 | 0.0006 | 0.0022 | 0.0011 | 0.0018 | 0.0010 |
| 20 | 0.0572 | 0.0098 | 0.0054 | 0.0035 | 0.0045 | 0.0034 | 0.0033 | 0.0021 | 0.0018 | 0.0009 |
| 21 | 0.3267 | 0.2359 | 0.0086 | 0.0038 | 0.0059 | 0.0020 | 0.0065 | 0.0021 | 0.0048 | 0.0022 |
| 22 | 0.1506 | 0.0930 | 0.0042 | 0.0019 | 0.0040 | 0.0018 | 0.0094 | 0.0043 | 0.0084 | 0.0045 |
| 24 | 0.2099 | 0.1062 | 0.0076 | 0.0024 | 0.0051 | 0.0022 | 0.0065 | 0.0014 | 0.0054 | 0.0011 |
| 25 | 0.1429 | 0.0034 | 0.0537 | 0.0056 | 0.0314 | 0.0025 | 0.0096 | 0.0015 | 0.0037 | 0.0002 |
| 26 | 0.2926 | 0.1358 | 0.0064 | 0.0009 | 0.0060 | 0.0015 | 0.0088 | 0.0018 | 0.0075 | 0.0021 |
| 27 | 0.0742 | 0.0341 | 0.0122 | 0.0028 | 0.0063 | 0.0016 | 0.0034 | 0.0007 | 0.0030 | 0.0005 |
| 28 | 0.2527 | 0.1684 | 0.0039 | 0.0008 | 0.0031 | 0.0013 | 0.0062 | 0.0033 | 0.0049 | 0.0025 |
| 29 | 0.2185 | 0.0904 | 0.0068 | 0.0015 | 0.0050 | 0.0016 | 0.0127 | 0.0034 | 0.0140 | 0.0037 |
| 30 | 0.0884 | 0.0117 | 0.0073 | 0.0010 | 0.0054 | 0.0009 | 0.0037 | 0.0009 | 0.0019 | 0.0006 |

**Table S9. Mean (and *SD*) DTW distance over right region electrodes (F8, FC6, T8) split by dyad and frequency bandwidth.**

| Group | Delta mean | Delta *SD* | Theta mean | Theta *SD* | Alpha mean | Alpha *SD* | Beta mean | Beta *SD* | Gamma mean | Gamma *SD* |
| --- | --- | --- | --- | --- | --- | --- | --- | --- | --- | --- |
| 1 | 0.0800 | 0.0283 | 0.0040 | 0.0012 | 0.0031 | 0.0007 | 0.0059 | 0.0016 | 0.0042 | 0.0014 |
| 3 | 0.2840 | 0.0897 | 0.0058 | 0.0016 | 0.0053 | 0.0021 | 0.0082 | 0.0037 | 0.0064 | 0.0033 |
| 6 | 0.2481 | 0.0663 | 0.0061 | 0.0009 | 0.0050 | 0.0015 | 0.0134 | 0.0046 | 0.0116 | 0.0039 |
| 7 | 0.1001 | 0.0591 | 0.0231 | 0.0059 | 0.0086 | 0.0009 | 0.0047 | 0.0010 | 0.0070 | 0.0008 |
| 8 | 0.1698 | 0.0542 | 0.0077 | 0.0062 | 0.0055 | 0.0033 | 0.0054 | 0.0025 | 0.0041 | 0.0019 |
| 9 | 0.3823 | 0.1527 | 0.0164 | 0.0019 | 0.0080 | 0.0013 | 0.0093 | 0.0008 | 0.0075 | 0.0009 |
| 10 | 0.0880 | 0.0278 | 0.0107 | 0.0036 | 0.0090 | 0.0031 | 0.0088 | 0.0029 | 0.0122 | 0.0032 |
| 11 | 0.4620 | 0.1627 | 0.0071 | 0.0031 | 0.0072 | 0.0020 | 0.0153 | 0.0022 | 0.0133 | 0.0027 |
| 12 | 0.1087 | 0.0175 | 0.0069 | 0.0021 | 0.0041 | 0.0012 | 0.0060 | 0.0025 | 0.0057 | 0.0025 |
| 13 | 0.1524 | 0.0465 | 0.0037 | 0.0014 | 0.0052 | 0.0021 | 0.0122 | 0.0055 | 0.0105 | 0.0053 |
| 15 | 0.0382 | 0.0000 | 0.0016 | 0.0000 | 0.0011 | 0.0000 | 0.0013 | 0.0000 | 0.0009 | 0.0000 |
| 16 | 0.1262 | 0.0427 | 0.0056 | 0.0008 | 0.0050 | 0.0006 | 0.0117 | 0.0025 | 0.0105 | 0.0022 |
| 17 | 0.1172 | 0.0773 | 0.0088 | 0.0038 | 0.0061 | 0.0023 | 0.0093 | 0.0031 | 0.0074 | 0.0020 |
| 18 | 0.0967 | 0.0131 | 0.0056 | 0.0023 | 0.0029 | 0.0006 | 0.0022 | 0.0006 | 0.0014 | 0.0004 |
| 20 | 0.0481 | 0.0102 | 0.0065 | 0.0048 | 0.0051 | 0.0037 | 0.0032 | 0.0018 | 0.0018 | 0.0006 |
| 21 | 0.3532 | 0.3517 | 0.0087 | 0.0032 | 0.0080 | 0.0015 | 0.0143 | 0.0086 | 0.0096 | 0.0053 |
| 22 | 0.2098 | 0.1149 | 0.0047 | 0.0022 | 0.0045 | 0.0018 | 0.0103 | 0.0048 | 0.0095 | 0.0061 |
| 24 | 0.1366 | 0.0577 | 0.0058 | 0.0022 | 0.0042 | 0.0004 | 0.0120 | 0.0022 | 0.0100 | 0.0017 |
| 25 | 0.1840 | 0.0956 | 0.0086 | 0.0039 | 0.0043 | 0.0023 | 0.0059 | 0.0033 | 0.0046 | 0.0029 |
| 26 | 0.1622 | 0.0976 | 0.0049 | 0.0012 | 0.0048 | 0.0027 | 0.0092 | 0.0041 | 0.0080 | 0.0032 |
| 27 | 0.0654 | 0.0262 | 0.0135 | 0.0032 | 0.0070 | 0.0019 | 0.0040 | 0.0009 | 0.0035 | 0.0007 |
| 28 | 0.1840 | 0.0930 | 0.0047 | 0.0010 | 0.0035 | 0.0010 | 0.0068 | 0.0024 | 0.0051 | 0.0017 |
| 29 | 0.1584 | 0.0963 | 0.0066 | 0.0022 | 0.0064 | 0.0032 | 0.0141 | 0.0076 | 0.0138 | 0.0076 |
| 30 | 0.0733 | 0.0157 | 0.0064 | 0.0022 | 0.0042 | 0.0016 | 0.0040 | 0.0013 | 0.0026 | 0.0009 |
